# Characterization of a de Novo Constitutional Balanced Translocation t (2;11)(q33.2;q23.2) with Break Point on the Human NBEAL1-GeneHo

**Published:** 2018

**Authors:** Javad KARIMZADHAGH, Soraya SALEHGARGARI, Mirdavood OMRANI

**Affiliations:** 1 Watson Genetic Laboratory, Tehran, Iran.; 2 Feto-maternal unit, Shohada Tajrish Hospital, Shahid Beheshti University of Medical Sciences, Tehran, Iran.; 3 Department of Medical Genetics, Faculty of Medicine, Shahid Beheshti University of Medical Sciences, Tehran, Iran.

**Keywords:** Balanced translocation, NBEAL1-Gene, ALS2-gene, ALS, FISH, BACs clone

## Abstract

Reciprocal balanced translocations associated with clinical features are very rare. This study reports cytogenetic and molecular cytogenetic findings in a 3-yr-old female patient with mild developmental retardation, slight hypotone with a de novo balanced 46, XX, t(2; 11) (q33; q23) translocation. Her parent attended private office at Tehran, Iran in 2013. G-banded chromosomes and FISH-Analysis were used to examine the patient's karyotype as well as her parents. FISH-probes prepared with specific RP11-BAC clones mapped near 2q33 and 11q23 regions were used to characterize the location of the breakpoints. One of the break points is located within the human NBEAL1-Gene locus on chromosome 2, suggesting a correlation between this gene disruption and the patient’s mild developmental retardation.

## Introduction

The Amyotrophic lateral sclerosis (ASL) is a progressive, neurodegenerative disease causing functionless of the motor upper neurons in the brain and lower motor neurons of the spinal cord systems and finally death from respiratory failure, usually within 3 to 5 yr of symptom onset. Over 90% of ALS are sporadic. Totally, 5%-10% is familial amyotrophic lateral sclerosis (FALS) but clinically indistinguishable from the sporadic form of the disease (1). Despite numerous efforts in the last decade, the basic pathophysiology of ALS remains unclear. Familial amyotrophic lateral sclerosis (FALS) is inherited as either autosomal dominant or autosomal recessive pattern. Recessive forms of ALS usually have juvenile onset. About 20% of FALS occur because of mutation found on SOD1 gene, but the causes of about 80% of adult-onset dominantly inherited FALS are not clear yet (2). One of the genes located on the critical region of type two ALS is NBEAL1 gene (ALS2CR17; Amyotrophic lateral sclerosis 2 chromosomal region candidate gene 17 proteins). The NBEAL1-Gene is a novel human neurobeachin-like 1 protein gene located on the chromosome two (2q33.2) and belongs to candidate genes for lateral sclerosis 2 (ALS2) with 25 Exons and two Beige/BEACH domains and 3 WD40 repeats. NBEAL1 gene spans about 73 kb of genomic DNA. The RT-PCR results showed that NBEAL1 is expressed mostly in the human brain, kidney, prostate, and testis. The significant rate of NBEAL1 gene expression in gliomas, compared to normal brain tissue, suggests a correlation with gliomas (3). However, there are no reported cases indicating the fact that, disruption of this gene could yield clinical phenotype. Although one of the causes of the gene disruption in neonates is autosomal reciprocal translocations, the frequency of this sort of translocation is very low (0.1%) (4). The majority of balanced translocations (96%) are not associated with clinical features (3). This is due to either translocation disrupting non-coding regions of genome or breakpoint is not on dose sensitive genes. Therefore, characterization of the genetic causes of the cases with de novo balanced translocation and clinical features is still favoured and followed by researchers, which could help to understand correlation mechanism of patient genotype and phenotype.

If carrier parent have a balanced rearrangement their offspring is in majority case normal. The risk for phenotypic abnormality is generally very low. Nonetheless, there are several reports for balanced translocation people with abnormal phenotypes, who has parent with the same balanced chromosomes but normal phenotype (5). 

The current study was an attempt to map the exact breakpoint on chromosome number two and eleven and to nominate and characterize the disrupted genes and their correlation with clinical findings on this patient.

## Case Report

**Fig 1:**
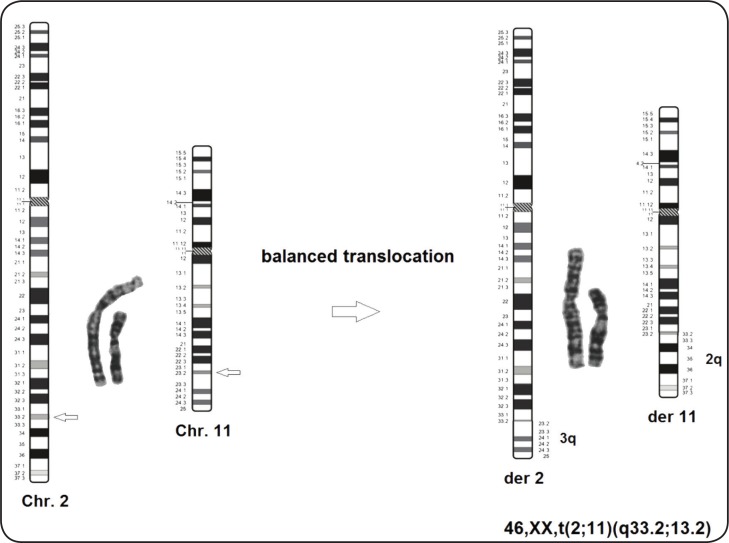
High-resolution GTG banding showed a reciprocal balanced translocation t(2;11)(q33.2;q23.2) in the fetus.

The case is a 3 yr old girl with mild developmental delay. Parents referred to private physicians’ clinics since the mother was over 35 during pregnancy in Tehran, Iran in 2013. The result of amniocentesis showed a balanced translocation in fetus with no other findings in ultrasound scans (46, XX, t(2; 11) (q33; q23) (Figure 1). Parents’ karyotypes were normal. After birth, the baby girl did not show any major or minor clinical anomalies and therefore was discharged from the hospital. After 3 yr, the girl showed some mild developmental delays, therefore, referred for further genetic analysis. Informed consent was taken from the parents.

1a: Translocated chromosomes 2 and 11 , 2 b: Normal chromosomes 2 and 11

Peripheral blood was collected for karyotype and DNA extraction. This procedure was carried out for both parents too. After locating, the primary breakpoint region in chromosome number two and eleven by high-resolution karyotype methods, 30 BACs clones were ordered for further analysis of the regions and then 21 was used for mapping the breakpoint.

**Preparing Locus Specific probes**

Chromosome preparations and high-resolution chromosomes were made from peripheral blood of the patient and her parents using standard methods. In order to map the breakpoint on the chromosome 2q33 and 11q23 regions, the following BACs (bacterial artificial chromosome) were ordered based on the information obtained from the genome browser part of http://genome.ucsc.edu/ (Table1).

**Table 1:** Used BACs, their position, and character

Name	Chromosome	Start	End	Length (kb)	Band
RP11-265N15	2	200002213	200178128	175.916	2q33.1
RP11-984A14	2	201531144	201699319	168.176	2q33.1
RP11-61B5	2	202446118	202606502	160.385	2q33.1
RP11-951H20	2	202897953	203067146	169.194	2q33.1
RP11-164J9	2	203452370	203611753	159.384	2q33.1-2q33.2
RP11-96E8	2	203558342	203715728	157.387	2q33.2
RP11-1133F11	2	203658771	203809833	151.063	2q33.2
RP11-771C13	2	203758666	203939481	180.816	2q33.2
RP11-963F8	2	203804972	204003029	198.058	2q33.2
RP11-916F5	2	208636277	208823743	187.467	2q33.3
RP11-14M16	11	112546742	112704340	157.599	11q23.1
RP11-1146P22	11	113500406	113657059	156.654	11q23.2
RP11-584C22	11	114601776	114812896	211.121	11q23.2
RP11-521L22	11	114820119	114986617	166.499	11q23.2
RP11-698I1	11	114914151	115091089	176.939	11q23.2
RP11-158B3	11	115064956	115220235	155.280	11q23.2
RP11-822K7	11	115141419	115327772	186.354	11q23.3
RP11-1081G12	11	114385681	114608391	222.711	11q23.2
RP11-984C17	11	115326382	115523057	196.676	11q23.2-11q23.3
RP11-2020	11	116250688	116416874	166.187	11q23.3
RP11-764D19	11	116994343	117176849	182.507	11q23.3

BACs DNA was labeled by Nick-Translation method using digoxigenin 11-dUTP (Roche) and Biotin (Invitrogen). DNA hybridization was performed as described in standard protocols. FISH slides were analyzed using a Zeiss Axioskop microscope with a cooled charged-coupled-device camera (Photometric) and then Imaging Meta system software was applied.

**Fig 2:**
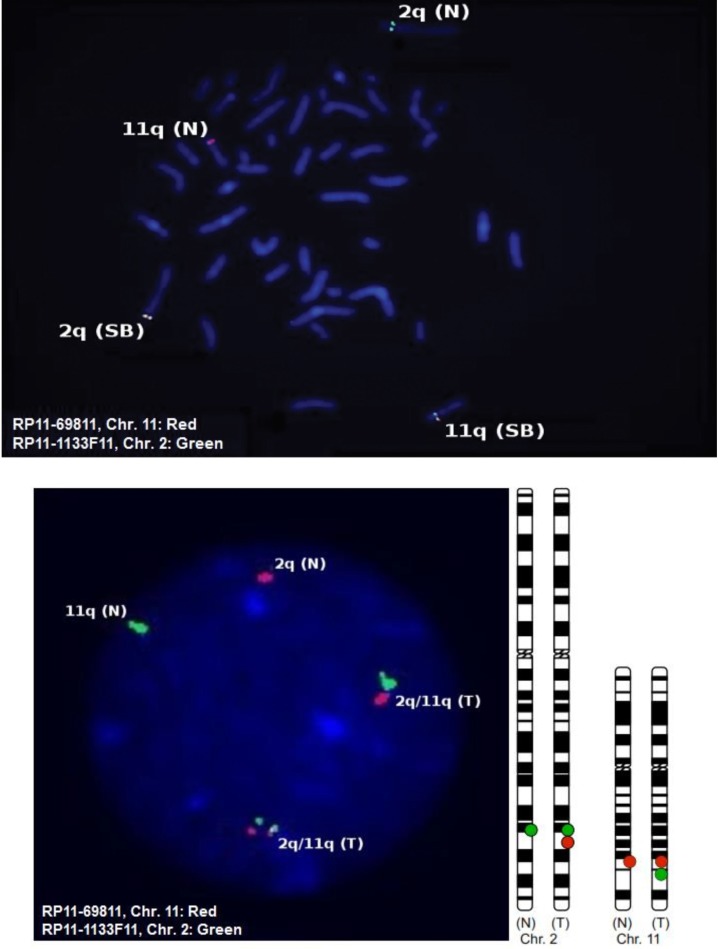
FISH signals in metaphase and interphase from peripheral blood and a combination of two FISH-probes from split BACs (SB). The split BAC of chromosome 2q (RP11-1133F11) was red labeled and the split BAC of chromosome 11q (RP11-698I1) was green labeled. In normal chromatid, only one signal either green (on Chr. 2) or red (on Chr. 11) were detected. In chromatid with translocation, two signals (Red/Green) or their combination (yellow) was detected.

Aminocytic analysis revealed a balanced translocation t (2q;11q) in the fetus. The mapping of breakpoint was carried out using locus-specific FISH probes (LSP) made on with about 21 BACs clones. All the selected FISH probes were hybridized with the long arm of chromosome number two except FISH probes made on BACs RP11-771C13, RP11-963F8, and RP11-916F5 that hybridized with long arm of chromosome 11. In addition, hybridization of BACs RP11-113FI1 was positive, but its signal was divided into two regions in the band 2q33.2 and chromosome 11. In chromosome number 11, all probes were hybridized and signals were obvious except for BACs probes RP11-158B3, Rp11-822K7, Rp11-984C17 and Rp11-2020 that hybridized with long arm of chromosome number 2. Rp11-69811signals was divided between long arm of chromosome number 2 and 11 (Figure 2). 

N=normal, T=translocated

## Discussion

**Fig 3:**
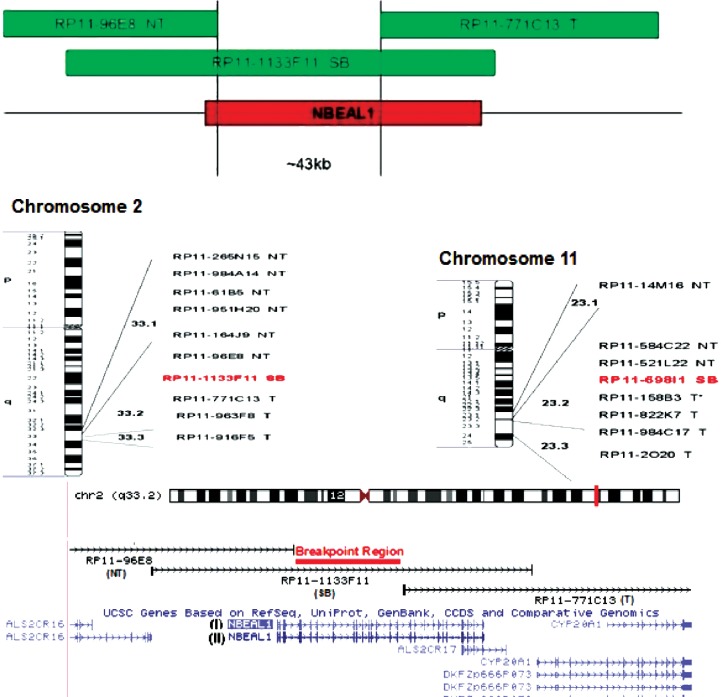
Some of the BACs clones were used for mapping the breakpoint region on chromosome 2 and how RP11-1133F11 probe overlap with NBEAL1 gene. NT=Not translocated, T=Translocated, SB=Spilt BACs

More than 90% of the de novo balanced translocation has no clinical impact since it occurs in the non-coding region of the genome or disrupts non-dose dependent genes. In the current study, a baby girl came to attention since she had mild developmental delay. Although her balanced translocation was noticed during her fetal period, her parents did not show any interest for further examination until she becomes 3 yr old and showed some symptoms. When locus-specific identifier (LSI) probes were designed to map the breakpoint between bands 2q33.1 and 2q33.3, all probes were hybridized, except the probe made from BAC RP11-771C13, RP11-963F8 and RP11-916F5 that hybridized with long arm of chromosome 11. In addition, hybridization of BAC RP11-113F11 was positive, but its signal was divided into two regions in the band 2q33.2 and chromosome 11. This clone contains about 157kb of chromosome 2q33.2 band. The clone start point is from 203558342bp and end is 203715728bp. When the LSI probe (Rp11-771C13) in the only 42938bp (about 43kb) downstream of this clone was used for hybridization purpose, FISH signal was negative on chromosome 2 but has positive signal on chromosome 11. Rp11-771C13 started from 203758666bp and ended in 203939481bp on the band 2q33.2. In the next step, the researcher chose an LSI probe that overlapped the distal part of Rp11-96E8 BAC and the proximal part of Rp11-771C13 BAC, in addition to covering 43 kb gap between these two probes. The start and end of the BAC Rp11-113F11 were 203658771-203809833bp, respectively. This probe was hybridized but its signals were divided between long arms of chromosome number 2 and 11. The Rp11-113F11 BAC probe signal may indicate that this signal was related to the 43 kb distance between these two BACs Rp11-96E8 and RP11-771C13 (Figure 3).

The application of blast for the sequence spanned in this 43kb region indicated that NBEAL1 gene was located in this region. It was for the first time that a correlation with the disruption of NBEAL1 gene and its consequence was shown. Unfortunately, after reporting this finding to the parents of the patient, they did not show interest for further cooperation and, consequently, it was not possible to sequence the NBEAL1 gene in the patient for its further analysis to identify its exact disruption point. 

In order to evaluate the breakpoint region on the long arm of chromosome 11 (11q23.2 band), among all used FISH probes only probe on BACs RP11-158B3, Rp11-822K7, Rp11-984C17, and Rp11-2020 did not hybridize with the expected region on chromosome 11 and instead hybridized with long arm of chromosome number 2. Rp11-69811 signals were divided between long arm of chromosome number 2 and 11Rp11-698I1. The start point of the BAC 114914151bp and the end is 115091089bp (176939bp). When this region sequence was blasted, no coding sequence was found which means this deletion is in the non-coding region of chromosome number 11 and has no effect on and contribution to the observed patient symptoms. 

**In conclusion,** despite this fact that more than 90% of the de novo balanced translocation has no clinical impact, in this special case it was shown that the phenotype of patient is because of her disrupted NBEAL1 gene. 
